# Recalculated HOMA-IR and Recorded Blood Pressure Changes in Treated Internal Medicine Patients: An Exploratory Retrospective Study

**DOI:** 10.3390/jcm15218496

**Published:** 2026-11-01

**Authors:** Camelia-Oana Mureșan, Bogdan Enache, Ionut-Dragos Capraru, Alexandra Enache, Ana-Maria Pah

**Affiliations:** 1 Institute of Legal Medicine Timișoara, 1A Cireșului Street, 300610 Timisoara, Romania; muresan.camelia@umft.ro (C.-O.M.); enache.alexandra@umft.ro (A.E.); 2 Ethics and Human Identification Research Center, “Victor Babeș” University of Medicine and Pharmacy of Timișoara, Eftimie Murgu Square 2, 300041 Timisoara, Romania; 3 Discipline of Forensic Medicine, Bioethics, Deontology, and Medical Law, Department of Neuroscience, “Victor Babeș” University of Medicine and Pharmacy of Timișoara, Eftimie Murgu Square 2, 300041 Timisoara, Romania; 4 Department of Cardiology, “Victor Babeș” University of Medicine and Pharmacy of Timișoara, Eftimie Murgu Square 2, 300041 Timisoara, Romania; anamaria.pah@umft.ro; 5 Department of Epidemiology, “Victor Babeș” University of Medicine and Pharmacy of Timișoara, Eftimie Murgu Square 2, 300041 Timisoara, Romania

**Keywords:** blood pressure, insulin resistance, HOMA-IR, antihypertensive treatment, omega-3 fatty acids, dietary advice, regression to the mean, retrospective cohort

## Abstract

**Background/Objectives**: In treated patients, concurrent metabolic and blood pressure (BP) changes are difficult to interpret without complete treatment histories. We examined the association between change in homeostasis model assessment of insulin resistance (HOMA-IR) and systolic BP (SBP), and described trajectories by prescribed management category. **Methods**: This exploratory retrospective cohort included 150 patients (132 inpatients and 18 outpatients at baseline), with visits labeled baseline, month 3, and month 6; exact dates were unavailable. Four nonrandomized categories were compared by baseline prescription, without adherence data. HOMA analyses excluded two participants with explicit insulin entries; complete-case regression used heteroscedasticity-consistent (HC3) confidence intervals (CIs). **Results**: The principal SBP coefficient was 2.20 mmHg per HOMA-IR doubling (95% CI −1.90 to 6.31; *n* = 99). Restrictions to no recorded baseline diabetes (*n* = 71) or no pharmacological glucose-lowering entry (*n* = 77) also yielded imprecise estimates. SBP decreased by 2.07–8.23 mmHg across prescribed categories, but adjusted contrasts were uncertain (global *p* = 0.743). The waist–SBP coefficient did not meet the multiplicity criterion (*q* = 0.107). Of 724 recorded BP values, 720 were multiples of 5 mmHg. **Conclusions**: The association between HOMA-IR change and recorded SBP change remained uncertain, and the efficacy of prescribed diet or omega-3 supplementation could not be assessed. The principal contribution of the study is to show how incompletely documented medication exposure, adherence, visit timing, and measurement conditions limit the interpretation of serial HOMA-IR and routine BP values in treated patients.

## 1. Introduction

Arterial hypertension reflects persistently elevated pressure within the systemic circulation and increases the risk of stroke, coronary disease, heart failure and kidney disease. A diagnosis depends on repeated, appropriately obtained measurements and clinical context; a single recorded value does not establish either the diagnosis or the adequacy of long-term control. The 2024 European Society of Cardiology guidance, cited together with its published corrections, places accurate measurement, cardiovascular risk, and tolerability at the center of management [1]. This distinction is especially relevant when longitudinal clinical records contain single visit-level values without out-of-office measurements. Such records can describe numerical trajectories, but stronger claims about sustained control require repeated, out-of-office assessment [2].

Excess adiposity provides a biologically plausible connection between metabolic abnormalities and elevated blood pressure (BP). Obesity can alter renal sodium handling, activate sympathetic and renin–angiotensin–aldosterone pathways, and influence pressure through renal compression and associated neurohumoral responses [3]. Insulin resistance denotes reduced responsiveness to insulin in tissues responsible for glucose and lipid regulation. In clinical studies, it is most often approximated by the homeostasis model assessment of insulin resistance (HOMA-IR), which is derived from a single fasting glucose–insulin pair and describes fasting glucose–insulin homeostasis, dominated by hepatic glucose output and basal insulin secretion; HOMA-IR is not equivalent to a direct measurement of peripheral, insulin-stimulated glucose disposal such as that obtained with the hyperinsulinemic–euglycemic clamp [4,5]. The clinical clustering of hyperinsulinemia, impaired glucose handling, and cardiovascular risk has long motivated investigations of insulin resistance as a contributor to hypertension [6]. Clamp-based research has shown reduced insulin sensitivity in selected patients with essential hypertension, even without obesity or glucose intolerance, indicating that body size alone does not capture the metabolic phenotype [7].

Vascular insulin signaling further connects metabolic and hemodynamic regulation. Impaired signaling in pathways involved in nitric oxide production can coexist with preserved signaling through pathways that favor vasoconstriction and cell growth. Endothelial dysfunction and insulin resistance can therefore reinforce one another, although their clinical expression varies with tissue, treatment and disease stage [8]. Adipose distribution also matters: body mass index (BMI) describes overall body size, whereas abdominal and ectopic fat may carry additional cardiometabolic information [9,10]. Waist circumference is a practical measure of abdominal size but does not quantify visceral fat directly or identify a specific renal or endothelial mechanism.

Longitudinal evidence supports studying the metabolic–blood pressure relationship while also illustrating the limits of transferring results between settings. The Bogalusa Heart Study examined how insulin resistance modifies the association between adiposity and incident hypertension; the KEIO study investigated insulin resistance and subsequent hypertension in middle-aged men [11,12]. These studies concern disease development over years. They do not directly estimate the association between simultaneous biomarker and blood pressure changes over a few months among patients receiving clinical care. A small weight-loss study using a glucose clamp investigated changes in insulin sensitivity alongside blood pressure, and the POUNDS Lost analysis considered temporal and mediation relationships during a structured two-year dietary intervention [13,14]. Their intervention content and measurement schedules differ from nonrandomized hospital records.

Lifestyle research provides another reason to distinguish average blood pressure change from its individual-level metabolic correlates. Dietary Approaches to Stop Hypertension (DASH) and DASH-Sodium demonstrated blood pressure effects of specified dietary patterns and sodium intake. PREMIER and ENCORE evaluated more comprehensive behavioral strategies [15,16,17,18]. A meta-analysis of randomized weight-reduction trials also supports an effect of weight loss on blood pressure [19]. These findings establish a substantial existing literature. They do not permit the effects observed in those trials to be assigned to a routinely prescribed dietary category or to an incompletely documented medication change.

Concurrent treatment adjustment creates a clinically relevant gap between the established association of insulin resistance with incident hypertension and the interpretation of serial metabolic measurements in treated patients. Baseline risk-factor associations and structured intervention trials do not establish whether short-term change in a fasting glucose–insulin index contains interpretable information about recorded BP change during routine care [6,7,11,12,13,14,15,16,17,18,19]. Antihypertensive titration, other metabolic treatment, and measurement conditions may obscure or mimic such a relationship. A retrospective analysis in this setting can characterize the uncertainty of an observed association, but cannot isolate the physiological contribution of insulin resistance from treatment effects.

A related evidence gap concerns the trajectories associated with routinely prescribed dietary advice and omega-3 supplementation alongside usual care: trial estimates concern defined interventions, whereas clinical records without adherence measurements capture recommendations. We therefore aimed, in an exploratory retrospective cohort, to estimate the association between change in recalculated HOMA-IR and recorded SBP change, to describe BP and metabolic trajectories by prescribed management category, and to document the features of routine records that constrain such interpretation. The study was not designed to establish treatment efficacy or to validate HOMA-IR as a monitoring biomarker; its findings are intended to inform the design of prospective studies.

## 2. Materials and Methods

### 2.1. Design, Setting and Relationship to the Companion Analysis

We analyzed longitudinal clinical records of 150 adults from the Department of Internal Medicine II, CFR Hospital, Timișoara, Romania, covering 1 October 2025 to 31 July 2026. Assessments were labeled baseline, month 3, and month 6, without random allocation. At baseline, 132 patients (88.0%) had an inpatient observation-chart number and 18 (12.0%) were evaluated in the outpatient/day-clinic setting. Reasons for individual admissions were not available in the analytical records. Recorded baseline diagnoses included hypertension (125/149), diabetes (40/149), and impaired glucose tolerance (21/149); these diagnoses do not establish the reason for hospitalization or clinical stability. Acute systemic inflammatory or infectious disease was an exclusion criterion. Table S7 describes the baseline setting. Reporting follows the Strengthening the Reporting of Observational Studies in Epidemiology (STROBE) principles [20].

This manuscript uses the same CFR cohort as a companion manuscript in preparation on recorded oxidative change and carotid wall thickness. The principal outcomes, regression analyses, and figures in the present manuscript concern blood pressure and metabolic change. Baseline clinical characteristics and some contextual variables are necessarily shared. Because both analyses derive from the same patients, they do not constitute independent cohorts, and this overlap is reported here in full.

Clinical information was extracted retrospectively from electronic records and paper charts. Index evaluations took place up to 31 January 2026 and follow-up evaluations up to 31 July 2026, with intended follow-up approximately 3 and 6 months after baseline. Exact visit dates and allowable windows were unavailable, so actual elapsed intervals could not be established. Month 3 and month 6 therefore denote source visit labels; no annualized change or common-time slope was estimated.

The target sampling frame comprised all consecutive adult inpatients and outpatients aged ≥18 years admitted with internal medicine conditions (primarily essential hypertension, metabolic syndrome, or type 2 diabetes mellitus). To audit selection bias, baseline logs of 210 consecutive patients admitted to or evaluated in Department II during the recruitment period were reviewed against eligibility criteria. Sixty patients were excluded based on a hierarchical, mutually exclusive decision rule: acute systemic inflammatory or infectious diseases at baseline (*n* = 18), active malignancy (*n* = 8), severe renal impairment with estimated glomerular filtration rate (eGFR) < 30 mL/min/1.73 m^2^ (*n* = 12), lack of baseline metabolic/HOMA-IR testing (*n* = 14), and no attendance at any follow-up visit after the index admission (*n* = 8). The remaining 150 unique patients constituted the primary source cohort. Because the last exclusion criterion depended on post-baseline attendance, it is acknowledged as a potential source of selection bias (Section 4.4). Missing individual visits or measurements among the 150 included patients (e.g., paired blood pressure in 108/150) were retained as missing within the source cohort and evaluated separately (Table S2).

### 2.2. Ethics and Consent

This retrospective observational study was conducted in accordance with the Declaration of Helsinki. Ethical approval was granted by the Ethics Council of CF Clinical Hospital Timișoara, Romania (approval No. 7110, dated 10 September 2026; Principal Investigator: Assoc. Prof. Camelia-Oana Mureșan, MD, PhD). The approval authorized the retrospective extraction, anonymization, and research processing of pre-existing inpatient and outpatient medical records from 1 October 2025 to 31 July 2026. All data extraction and research analyses were initiated after obtaining ethical clearance in September 2026. Written informed consent was obtained from all patients at admission, authorizing the anonymized use of their clinical and laboratory data for research. Confidentiality was strictly maintained throughout.

### 2.3. Blood Pressure Measurements and Outcomes

The principal outcome was recorded systolic blood pressure (SBP) at the month-6-labeled visit minus baseline SBP, in mmHg. Diastolic blood pressure (DBP) change was a companion outcome. Negative differences indicate lower recorded follow-up values. Participants were the unit of inference. The metabolic objective was to estimate the association between change in recalculated HOMA-IR and SBP change; a second objective was to describe BP and metabolic changes across the four prescribed management categories.

The department reports a routine protocol using an oscillometric upper-arm monitor (Omron M3/HEM-7154-E), a 5-min rest and three consecutive readings, with the mean of the second and third recorded. Only visit-level chart entries were available; the analytical dataset did not include individual readings, device output, or per-visit protocol compliance. Of 724 SBP and DBP entries, 720 (99.4%) were multiples of 5 mmHg and 512 (70.7%) ended in zero (Table S8), indicating marked terminal-digit preference whose origin (rounding, measurement method, or transcription) cannot be established. Values were analyzed as recorded. Baseline measurements were obtained during inpatient or day-clinic evaluation, whereas follow-up measurements were obtained in the outpatient setting.

The reported departmental protocol allowed manual auscultatory measurement for clinically significant arrhythmias, averaging two readings. The analytical records did not identify the method used for each participant or visit, so manual and oscillometric measurements could not be separated.

For descriptive threshold transitions, values were classified as below 140/90 mmHg only when SBP was <140 and DBP was <90; below 130/80 required both SBP < 130 and DBP < 80. Equality counted as not below the threshold. These operational classifications describe recorded values and are not diagnoses of remission, sustained control, or individualized guideline-target attainment. Baseline-to-six-month transitions used only patients with both components at both visits. The three-visit trajectory analysis required all three values of the relevant pressure component and used the same patients at every displayed time point.

### 2.4. Metabolic and Anthropometric Measurements

HOMA-IR was recalculated as fasting glucose (mg/dL) × fasting insulin (μU/mL)/405 [4]. This approximation describes fasting glucose–insulin homeostasis, predominantly hepatic glucose regulation together with basal insulin secretion and clearance; it is not a direct measure of peripheral insulin-stimulated glucose disposal [5]. For revised HOMA-specific summaries and models, participants with an explicit insulin entry at any visit were excluded. The original all-record SBP model is retained only as a numerical reference in Table S1.

According to the reported clinical protocol, venous samples were obtained in the morning after an overnight fast of 10–12 h. Glucose was measured by a hexokinase method on a Roche Cobas c module and insulin by electrochemiluminescence immunoassay on a Cobas e module, reported in mg/dL and μU/mL, respectively. Individual fasting duration, the timing of the last medication dose, and assay quality-control records were not available, so the fasting steady-state conditions underlying HOMA could not be verified for each observation.

HOMA assumes fasting steady-state glucose and endogenous insulin [4,5]. Subcutaneous exogenous insulin bypasses portal delivery and first-pass hepatic extraction, and assay detection of insulin analogs varies; insulin-based HOMA estimates under these circumstances require validation that was unavailable here [5,21]. Two participants had an explicit insulin entry at one or more visits and were excluded from the revised HOMA analyses; this reviewer-directed change was made after the original analysis and is not presented as prespecified. A nonflagged record indicates no explicit entry, not verified insulin-naive status. Free-text diabetes treatment was classified by recorded drug class at each visit (Table S6). Because metformin, sulfonylureas, thiazolidinediones, incretin-based agents and sodium–glucose cotransporter-2 (SGLT2) inhibitors can influence glucose, insulin or both, and doses and dose timing were unavailable, restrictions to no baseline diabetes, no diabetes at any visit and no pharmacological glucose-lowering entry were given particular interpretive attention. Insulin-antibody status, C-peptide, and dynamic secretion tests were unavailable, so antibody-related assay interference could not be excluded [21].

The metabolic exposure was log2(month-6/baseline HOMA-IR), with baseline log2 HOMA-IR included in the model. A value of +1 represents doubling and −1 halving; only positive values were eligible. Separate anthropometric models used waist change per 5 cm or weight change per kg and the corresponding baseline measurement. The reported anthropometric protocol used a calibrated column scale/stadiometer, light clothing without shoes, and waist measurement at normal expiration midway between the lowest rib and iliac crest. Per-visit compliance could not be checked. Tumor necrosis factor-α (TNF-α) and interleukin-6 (IL-6) fields lacked documented units, detection limits, and zero coding (Table S5); they were therefore not used to diagnose inflammatory disease or as adjustment covariates.

### 2.5. Recorded Management and Medication Information

The four categories reflected the baseline clinical recommendation: control, usual clinical management (*n* = 39); alim, dietary advice (*n* = 37); omega, omega-3 supplementation (*n* = 37); and alim+omega, both (*n* = 37). Assignment followed physician discretion, and comparisons used the recorded baseline category regardless of unknown subsequent adherence. This is an observational comparison by prescribed category, not a randomized intention-to-treat estimate, and cannot separate the recommendation from confounding by indication, actual consumption, other care, or adherence. Category indicators were also entered into the metabolic–pressure models, with control as reference.

The reported dietary prescription followed Mediterranean principles, with a 500-kcal/day deficit for patients with BMI ≥ 25 kg/m^2^, sodium below 2 g/day and carbohydrate intake of approximately 45–50% of energy. The reported omega-3 prescription was 1000 mg of ethyl-ester product twice daily, each capsule containing at least 460 mg eicosapentaenoic acid (EPA) and 380 mg docosahexaenoic acid (DHA), corresponding to approximately 1680 mg EPA+DHA/day. These details describe the prescribed regimen; achieved dietary intake, sodium intake, and supplement consumption were not measured.

Recommendations were intended to continue through the month-6-labeled visit. Concurrent antihypertensive and metabolic treatment was adjusted at the treating physician’s discretion and was neither standardized nor held constant.

Five cardiovascular medication fields were available at each visit under headings corresponding to beta-blockers, angiotensin-converting enzyme (ACE) inhibitors, calcium-channel blockers, diuretics, and angiotensin receptor blockers. Text entries, inconsistent spelling, and some apparent class-placement errors prevented reliable reconstruction of a pharmacological regimen. For limited sensitivity analyses, each field was coded as occupied when it contained a nonblank entry other than numeric/text zero; blank entries remained missing, and a count was computed only when all five fields were documented. This variable counts occupied source fields, not validated drugs, drug classes, or doses; no medication names were moved between columns and no doses were inferred. Recorded occupancy of each antihypertensive and lipid-lowering heading at each visit is reported in Table S6.

### 2.6. Data Checks and Incomplete Records

Analyses used available records for each specified outcome and covariate set, without replacing missing values. The source contains 150 patients; this number is not the effective sample size of every analysis. Availability was tabulated by recorded category. Baseline characteristics of patients with paired blood pressure versus other source records were compared descriptively.

Discordance between the glucose mg/dL and mmol/L fields was flagged when |glucose mg/dL/18 − glucose mmol/L| exceeded 0.2 mmol/L. The principal HOMA calculation used the mg/dL field; a sensitivity analysis excluded participants with a flagged baseline or month-six glucose pair. Stored and recalculated HOMA disagreement was flagged when the absolute difference exceeded the larger of 0.15 units or 10% of recalculated HOMA. The stored index was used only in a separate sensitivity analysis. Implausibly low stored BMI entries of 2.7 and 2.5 were excluded from their affected visit-specific BMI summaries and were not corrected to assumed values. Source flags are not proof of the direction or magnitude of an error. Numeric text with a decimal comma was parsed as a decimal value; no missing glucose or insulin value was inferred. An explicit insulin flag was defined by the text fragment “insulin” in any of the three diabetes-treatment fields. All revised HOMA-specific summaries and models exclude flagged records. Baseline no-recorded-diabetes required a diabetes code of zero; the stricter restriction required zero at each of the three visits and excluded missing codes.

Complete-case analyses can lose precision and introduce selection bias when follow-up or measurement availability depends on clinical status, treatment, or unobserved behavior [22]. Of 150 source records, 42 lacked paired SBP, seven further records lacked paired glucose–insulin inputs, and two otherwise complete records had explicit insulin exposure, leaving 99 (66.0%) in the revised principal model. The last two exclusions concern HOMA interpretability rather than missing data. Table S4 compares the 99 analyzed with the 51 other records, and Table S2 describes paired-pressure availability (108 versus 42). Reasons for missing follow-up measurements were not documented, so the direction of selection bias is unknown, and observed baseline similarity cannot establish random missingness. No imputation was performed, because imputing numerical variables would not restore an undocumented treatment history or measurement protocol. Different denominators limit comparisons between exposure models.

### 2.7. Statistical Analysis

Continuous variables are summarized as mean ± standard deviation (SD) or median [interquartile range (IQR)], with denominators. Paired changes are follow-up minus baseline. Their mean-change intervals are percentile bootstrap 95% confidence intervals (CIs) based on 10,000 participant resamples, seed 82110. Two-sided Wilcoxon signed-rank tests summarize paired distributional changes; the rank test and the bootstrap interval for mean change address different estimands. Descriptive threshold transitions use the exact two-sided McNemar test, implemented as a binomial test of the discordant pairs.

Baseline characteristics were compared across the four categories descriptively, using Kruskal–Wallis tests for continuous variables and chi-square tests for proportions. Baseline-to-month-six changes in SBP, DBP, log_2_ HOMA-IR ratio, waist circumference and body weight were summarized by category as means with participant-bootstrap 95% CIs. Between-category differences were estimated by linear regression of each change on category indicators (control as reference), the corresponding baseline value, age per decade and sex, with heteroscedasticity-consistent (HC3) standard errors; a Wald test assessed any difference among the four categories.

The revised principal model regressed SBP change on log2 HOMA ratio, baseline SBP, baseline log2 HOMA-IR, age per decade, sex, and three category indicators, excluding explicit insulin exposure. Separate models related HOMA change, waist change, and weight change to SBP and DBP. HOMA models used the insulin-exclusion subset; anthropometric models used their available complete cases. HC3 standard errors were combined with Student t intervals using residual degrees of freedom [23]. Baseline adjustment does not eliminate measurement error, confounding, or regression to the mean [24,25].

Benjamini–Hochberg *q* values were calculated within five explicitly defined exploratory families: six metabolic–pressure regression coefficients; five overall paired changes; six interval contrasts in the three-visit SBP/DBP subset; two threshold-transition tests; and the 15 between-category contrasts (three contrasts with control for each of five outcomes) [26]. Sensitivity models were interpreted by their estimates and intervals, without a separate claim of statistical discovery. Confidence intervals are pointwise rather than simultaneous. No data-derived responder cutoff was used.

All analytical choices were exploratory and made after data access. Following peer review, the principal HOMA analysis was revised to exclude explicit insulin entries; the original 101-patient coefficient remains a labeled reference in Table S1. Sensitivities based on the revised analysis added BMI and diabetes; excluded glucose-unit discrepancies; restricted to no baseline diabetes or no diabetes at any visit; used stored HOMA; required complete three-visit pressure and HOMA; omitted category indicators; added baseline occupied antihypertensive fields, with or without their change; excluded any pharmacological glucose-lowering entry; restricted baseline setting to inpatients; or adjusted for baseline setting. Medication-change adjustment conditions on a follow-up variable and can introduce overadjustment or collider bias, so it is a documentation sensitivity rather than a causal analysis. The relationship between baseline SBP and SBP change was summarized descriptively; mathematical coupling and treatment responses limit its use as evidence of regression to the mean.

The sample consisted of available retrospective records. No prospective sample-size calculation or sample-size test was documented or performed, so no such test can be reported. Achieved confidence-interval precision is presented instead of observed post hoc power. For context, given the achieved standard error of the revised principal coefficient (approximately 2.07 mmHg), the minimum association detectable with 80% power at two-sided α = 0.05 was approximately 5.9 mmHg per doubling of HOMA-IR; smaller associations could not be reliably detected. Revised calculations used Python (version 3.12.7; Python Software Foundation, Wilmington, DE, USA) with NumPy (version 2.1.3), pandas (version 2.2.3), and SciPy (version 1.14.1), ordinary least squares, and an explicit HC3 covariance calculation. Global category tests used an HC3 Wald F statistic with three numerator and residual denominator degrees of freedom. Results are exploratory, and nonsignificance does not establish absence of association or equivalence [27].

## 3. Results

### 3.1. Cohort and Analytical Availability

The source contained 150 patients, with a mean age of 54.99 ± 10.43 years; 71 (47.3%) were women. Recorded hypertension was present in 125/149 (83.9%) and diabetes in 40/149 (26.8%) patients with those fields documented. Baseline BMI averaged 33.98 ± 4.81 kg/m^2^ among 148 usable records, and baseline waist circumference was 110.30 ± 11.24 cm among 149. These characteristics describe a cohort with substantial recorded cardiometabolic burden. At baseline, 132 patients (88.0%) were hospitalized, and 18 (12.0%) were outpatients; outpatients were younger (48.28 vs. 55.90 years) and had higher baseline SBP (151.76 vs. 140.98 mmHg) (Table S7). At least one antihypertensive medication field was occupied at baseline in 109/139 patients with complete fields (78.4%) and at least two in 73/139 (52.5%). Pharmacological glucose-lowering treatment was recorded at baseline in 26/149 patients (classes not mutually exclusive; Table S6): metformin in 20, a sulfonylurea in 10, a thiazolidinedione in one, insulin in one, and another entry in one; 15 were managed by diet alone. No incretin-based therapy or SGLT2 inhibitor was recorded at any visit. Among 108 patients with the field documented at baseline and month six, recorded drug classes changed in four (Table S6).

Paired SBP and DBP were available for 108/150 patients (72.0%). After seven further exclusions for missing glucose–insulin pairs and two for explicit insulin exposure, the revised principal HOMA–SBP model included 99/150 (66.0%). Its category counts were 21 control, 27 diet, 23 omega-3, and 28 combined. Waist and weight models included 105 and 100 patients. Three-visit pressure summaries used 88 complete records for each component. Table 1 describes the cohort; Table S4 separates the sequential reasons for non-inclusion. Baseline HOMA summaries also exclude the two participants with any explicit insulin entry.

Observed baseline differences between records included in and excluded from the revised principal model were small (Table S4): mean age 55.53 versus 53.94 years (standardized mean difference [SMD] 0.151), baseline SBP 142.98 versus 140.70 mmHg (SMD 0.110), and BMI 34.11 versus 33.74 kg/m^2^ (SMD 0.076). Mean recalculated baseline HOMA-IR was 4.28 versus 3.80 (SMD 0.169); the latter group includes the two insulin-exposed records for this inclusion audit only. All reported SMDs were below 0.2. Paired-pressure availability (108 versus 42) is compared in Table S2.

### 3.2. Characteristics and Trajectories by Prescribed Management Category

The nonrandomized categories differed at baseline (Table 2). Control patients were older (60.10 years versus 51.59–55.76 years; *p* = 0.002) and more often had an occupied antihypertensive field (96.9% versus 55.6% in the combined category; *p* < 0.001). Recorded hypertension was least frequent in the combined category (64.9%; *p* = 0.003); weight also differed (*p* = 0.040). No clear baseline difference was detected for SBP, DBP, waist, or recalculated HOMA-IR.

Between baseline and the month-6-labeled visit, mean SBP decreased by 8.12 mmHg in control (*n* = 24), 2.07 in diet (*n* = 29), 8.12 in omega-3 (*n* = 24), and 8.23 in combined (*n* = 31) (Table 3). Baseline-, age- and sex-adjusted differences from control were 4.00 mmHg (95% CI −4.87 to 12.87), 1.27 (−9.68 to 12.22) and 0.74 (−8.97 to 10.45), respectively (global *p* = 0.743). DBP, log2 HOMA ratio, waist and weight contrasts were also imprecise (global *p* = 0.584, 0.881, 0.700 and 0.883). All 15 adjusted contrasts had *q* = 0.980. All intervals included clinically relevant differences in either direction.

### 3.3. Timing of Observed Blood Pressure Change

In the fixed 88-patient three-visit subset, mean SBP was 141.31 ± 18.99 mmHg at baseline, 133.41 ± 17.05 at month three, and 135.57 ± 16.05 at month six. Mean change was −7.90 mmHg from baseline to month three (95% CI −12.33 to −3.58; *q* = 0.002) and +2.16 mmHg from month three to month six (95% CI −1.68 to 6.05; *q* = 0.469). Baseline-to-six-month mean change was −5.74 mmHg (95% CI −10.00 to −1.57; *q* = 0.019). Thus, the observed mean reduction occurred mainly before the intermediate visit; the second-interval estimate was compatible with both a modest further decrease and an increase.

DBP in the same-sized complete subset averaged 85.00 ± 10.56, 79.94 ± 10.95 and 80.41 ± 9.97 mmHg. Its mean change was −5.06 mmHg during the first interval (95% CI −7.56 to −2.61; *q* = 0.001) and +0.47 mmHg during the second (95% CI −2.00 to 2.95; *q* = 0.863). Table 4 and Figure 1 summarize the common-subset trajectories.

In the larger 108-patient paired-pressure subset, mean SBP change was −6.53 mmHg (95% CI −10.55 to −2.73; *q* = 0.008) and DBP change −5.96 mmHg (95% CI −8.14 to −3.81; *q* < 0.001). Revised paired HOMA change was −0.19 units (*n* = 99); waist change was −0.98 cm (*n* = 105) and weight change −1.04 kg (*n* = 100) (Table S3). These estimates derive from different available subsets.

SBP change was inversely correlated with baseline SBP (Spearman ρ = −0.684; *p* < 0.001; *n* = 108). Mean change was −16.89 mmHg in patients with baseline SBP ≥140 (*n* = 61) and +6.91 mmHg in those below 140 (*n* = 47). It was −4.41 mmHg among 94 patients hospitalized at baseline and −20.71 among 14 baseline outpatients (Mann–Whitney *p* = 0.028), whose baseline SBP was higher. Because baseline SBP is part of the change score, this correlation is partly mathematical; possible explanations are considered in Section 4.4.

### 3.4. Revised HOMA Association and Anthropometric Comparisons

After exclusion of explicit insulin exposure, the adjusted SBP coefficient was 2.20 mmHg per doubling of HOMA-IR (95% CI −1.90 to 6.31; *p* = 0.289; q = 0.434; *n* = 99). The interval permits associations in either direction. The model was adjusted for baseline SBP, baseline log2 HOMA-IR, age, sex, and prescribed category. The original numerical coefficient including the two insulin-exposed records was 1.76 mmHg (95% CI −2.19 to 5.71; *n* = 101) and is shown for reference only (Table S1).

The revised DBP–HOMA coefficient was −0.47 mmHg per doubling (95% CI −3.02 to 2.08; *p* = 0.718; *q* = 0.718; *n* = 99). In the separate waist model, the SBP coefficient was 3.22 mmHg per 5 cm (95% CI 0.57 to 5.87; *p* = 0.018; *q* = 0.107; *n* = 105). Its pointwise interval excluded zero, but the waist result did not meet the multiplicity criterion. The waist–DBP coefficient was 1.14 mmHg per 5 cm (95% CI −0.88 to 3.15; *q* = 0.434).

Weight-change coefficients were 0.56 mmHg/kg for SBP (95% CI −0.23 to 1.35; *q* = 0.434) and 0.13 for DBP (95% CI −0.35 to 0.61; *q* = 0.705). Table 5 reports all six coefficients and the recomputed *q* values. Figure 2 shows unadjusted individual changes.

### 3.5. Treatment Documentation and Sensitivity Estimates

All five antihypertensive fields were documented in 139 baseline, 104 month-3, and 105 month-6 records. The median occupied-field count was 2 [1,2] at every visit. Among 102 patients with complete baseline and month-6 fields, counts decreased in 6, remained unchanged in 82, and increased in 14. TNF-α and IL-6 entries were available at baseline in 84/99 and 83/99 revised principal-model records, respectively. Because units and zero coding were unverified, these markers were not used for adjustment (Table S5).

Adding baseline occupied medication fields to the revised model yielded a coefficient of 1.87 mmHg per doubling (95% CI −2.79 to 6.54; *n* = 95); adding their change yielded 2.06 (−2.65 to 6.77; *n* = 95).

Restrictions addressing HOMA interpretability were emphasized independently of statistical significance. With no diabetes recorded at baseline, the coefficient was 2.32 mmHg per doubling (95% CI −2.36 to 7.00; *p* = 0.325; *n* = 71). Requiring no diabetes recorded at all three visits gave 0.73 (−5.99 to 7.46; *n* = 55). Excluding any pharmacological glucose-lowering entry gave 2.20 (−2.91 to 7.31; *n* = 77). All intervals remained compatible with associations in either direction.

Additional sensitivities were similarly imprecise: adding BMI and diabetes yielded 2.18 mmHg (95% CI −1.86 to 6.23; *n* = 97), excluding glucose-unit discrepancies yielded 2.19 (−3.04 to 7.42; *n* = 84), restricting to baseline inpatients yielded 2.12 (−2.26 to 6.50; *n* = 85), and adjusting for baseline setting yielded 2.02 (−2.06 to 6.09; *n* = 99). Table S1 and Figure 3 report all revised specifications, including stored-HOMA and complete-three-visit analyses. Across all revised specifications, coefficients ranged from 0.73 to 2.32 mmHg per doubling, and every 95% CI included zero.

### 3.6. Transitions Across Descriptive Pressure Thresholds

Among 108 patients with both pressure components at baseline and month six, 42 (38.9%) were below 140/90 mmHg at baseline and 62 (57.4%) at month six. Thirty-one moved from not below to below the threshold, 11 moved in the opposite direction, 31 remained below, and 35 remained not below. Exact McNemar *p* was 0.003, and *q* was 0.006 across the two threshold tests.

For the stricter operational threshold, 14 (13.0%) were below 130/80 at baseline and 26 (24.1%) at month six. Twenty-one crossed downward and nine upward; only five remained below at both visits. The exact *p* and *q* values were 0.043. Table 6 reports all transitions.

## 4. Discussion

### 4.1. Principal Findings and Clinical Contribution

The revised estimate after excluding recorded insulin exposure was 2.20 mmHg per doubling of HOMA-IR, with a 95% CI from −1.90 to 6.31. Its direction and magnitude remain uncertain. Restrictions to no recorded diabetes or no pharmacological glucose-lowering entry did not resolve this uncertainty. Category contrasts also allowed meaningful differences in either direction. The nominal waist–SBP result did not meet the multiple-comparison criterion. Lower mean recorded BP at follow-up is a descriptive observation that cannot be attributed to a specific recommendation, treatment, or metabolic mechanism.

The study therefore provides an exploratory estimate in a heterogeneous routine-care population rather than a new test of the established relationship between insulin resistance and hypertension [6,7,11,12]. A nonsignificant coefficient cannot establish that metabolic change is unrelated to BP response or that HOMA-IR monitoring is ineffective.

In our view, the principal clinical contribution of the study lies less in identifying a definitive metabolic–BP relationship than in demonstrating, with routinely collected records, the interpretative challenges of using serial HOMA-IR and routine BP measurements in treated patients. In this cohort, recorded insulin exposure made some HOMA-IR values uninterpretable; exposure to other glucose-lowering drugs was incompletely documented, and doses and dose timing were unavailable; antihypertensive regimens could not be reconstructed from free-text fields; adherence was not measured; visit intervals were known only by label; and 720 of 724 BP entries were multiples of 5 mmHg. These documentation gaps limit interpretation of the association, which was estimated imprecisely in this cohort. For clinicians, the practical implication is that a between-visit change in HOMA-IR should not be used to explain or predict a patient’s BP response unless insulin and other glucose-lowering treatment, antihypertensive changes, adherence, sampling conditions and the BP measurement method are documented. For investigators, the same gaps define the minimum documentation required before routinely collected data can address this question (Section 4.5).

### 4.2. Trajectories by Prescribed Management Category

Adjusted BP and metabolic trajectories were uncertain across prescribed categories. The SBP intervals for comparisons with control extended approximately from −10 to +13 mmHg and included reductions of magnitudes reported in controlled dietary and omega-3 trials [15,16,17,18,19,28]. The results therefore neither establish a benefit of the recommendations nor demonstrate equivalence. Comparisons concern baseline prescriptions; without adherence measurements, they cannot identify the effect of the diet or supplement actually consumed.

The older age and more frequent antihypertensive documentation in control patients illustrate confounding by indication. Covariate adjustment cannot remove differences that were incompletely recorded. The common BP decrease across categories may reflect shared treatment changes, measurement circumstances, and regression to the mean. This design cannot separate those explanations.

### 4.3. Interpretation in Relation to Previous Evidence

Incident-hypertension cohorts, clamp-based weight-loss research and structured dietary trials address different populations, interventions and outcomes [11,12,13,14]. They should not be interpreted as directly estimating simultaneous HOMA and recorded BP changes in a heterogeneous treated cohort. Insulin-based HOMA combines fasting insulin action with secretion and clearance [4,5]. Population differences further complicate transportability: a meta-analysis of 74 cohorts found different relationships between insulin sensitivity and acute insulin response across African, European-ancestry, and East Asian groups [29]. These are population-level patterns, not individual biological classifications. This single-center Romanian cohort had no recorded ancestry characterization, C-peptide, or dynamic secretion tests, so no Romanian-specific secretory capacity can be inferred. A within-person ratio also does not remove treatment-related changes in secretion or clearance. The two 2026 reports on HOMA-related BP variability and prediction of insulin resistance from wearable and routine data provide contemporary context, but do not validate HOMA interpretation under uncertain insulin exposure or establish its use for monitoring BP response [30,31].

Controlled evidence supports BP effects of specified dietary patterns, sodium reduction, weight loss, exercise, reduced alcohol intake and omega-3 supplementation [15,16,17,18,19,28,32,33]. DiRECT illustrates the importance of contemporaneous prescribing information when interpreting metabolic and BP change during weight loss [34]. Randomized trials and meta-analyses establish cardiovascular benefits of BP lowering [35,36,37,38,39,40], but the present study measured neither cardiovascular events nor randomized treatment effects.

### 4.4. Strengths and Limitations

Strengths include source-based HOMA recalculation, explicit analytical denominators, declared multiplicity families, comparisons by prescribed category and reviewer-directed restrictions addressing insulin exposure. The substantial limitations are consolidated below.

First, antihypertensive therapy was neither standardized nor adequately reconstructed, which is the principal limitation. Physicians could change products or doses in response to the same BP and metabolic measurements under study. Occupied free-text fields cannot detect dose escalation, distinguish consistent drug classes, or verify adherence. Treatment can confound or modify metabolic–pressure associations, and its net contribution cannot be estimated here. Medication-field sensitivities (Section 3.5) cannot resolve this problem, and adjusting for a follow-up treatment proxy can itself introduce bias; no estimate describes BP change under constant treatment.

Second, measurement setting differed by visit and initial clinical status was incompletely characterized. Most baseline observations were inpatient, whereas all follow-up observations were outpatient. Admission-related stress, recovery, and recent treatment changes may affect BP, glucose, and insulin simultaneously. Baseline outpatient observations do not solve this problem because they were few and clinically different. Regression to the mean is also plausible [41], but the correlation of baseline BP with its own change partly reflects mathematical coupling and cannot quantify that mechanism. Neither baseline adjustment nor setting adjustment separates true physiological change from these influences.

Third, BP recording showed pronounced terminal-digit preference, inconsistent with unrounded oscillometric output, and rounding, manual measurement, and transcription practices cannot be distinguished retrospectively [2,42]. This coarsening may reduce precision, conceal small changes, and bias comparisons, particularly threshold transitions, if recording differed by setting or threshold. Individual readings and out-of-office measurements were unavailable.

Fourth, adherence to diet, supplements and medications were not measured, nor were achieved sodium intake, physical activity or alcohol intake. Comparisons by baseline prescription do not estimate efficacy. Nonadherence could attenuate contrasts, but in this observational setting its association with prognosis, switching and other care means that the direction of bias is uncertain.

Fifth, exact visit dates and actual intervals were unavailable, which prevents estimation of change per unit time and limits temporal interpretation of trajectories.

Sixth, excluding explicit insulin entries improves the revised HOMA analysis but does not exclude unrecorded exposure or verify fasting steady state. Other glucose-lowering drugs, beta-cell reserve, insulin clearance, and insulin antibodies can affect measured insulin and glucose, and doses, C-peptide, dynamic secretion, and antibody testing were unavailable. Restrictions excluding recorded diabetes or glucose-lowering drugs remain subject to incomplete ascertainment and do not certify insulin-naive status or normal physiology.

Seventh, prescribed categories were non-random, and actual reasons for hospitalization were not available. Recorded diagnoses do not replace admission indications or severity. Residual confounding by initial illness, treatment indication, and subsequent care is therefore likely.

Eighth, incomplete follow-up and eligibility selection limit generalizability. Only 99 of 150 source records entered the revised principal model (Section 2.6; Table S4), and eight screened patients without follow-up had been excluded before formation of the source cohort. Baseline comparisons cannot exclude selection on unobserved changes or indicate its direction.

Finally, analytical priorities and the revised exclusion rule were selected after data access; the effective sample was small relative to the covariates (maximum leverage 0.226), and no equivalence margin or clinical-utility validation was defined [27]. TNF-α and IL-6 had unverified units and zero coding, preventing a validated inflammation-adjusted interpretation. The cohort overlaps a companion analysis and is not an independent replication.

### 4.5. Implications for Future Research

A confirmatory study would require prospective registration, exact visit dates, standardized BP measurement with stored readings and out-of-office assessment, documented medication names, doses and changes, and adherence measurements. HOMA interpretation would require verified absence of exogenous insulin and adequate characterization of fasting conditions and other treatment, with C-peptide or direct insulin-sensitivity assessment where appropriate. Randomization would be required to estimate the efficacy of diet or omega-3 supplementation alongside usual care.

## 5. Conclusions

In this exploratory retrospective cohort, the association between change in recalculated HOMA-IR and recorded SBP change remained uncertain after excluding explicit insulin exposure and in further restrictions addressing recorded diabetes and glucose-lowering treatment. Differences between prescribed management categories were imprecise, the waist–SBP result did not meet the multiplicity criterion, and lower mean BP at follow-up cannot be attributed to a particular treatment or metabolic improvement. The data therefore cannot support serial HOMA-IR for monitoring BP response or establish the efficacy of the recorded recommendations. Their principal clinical value is to show that, in treated patients, serial HOMA-IR and routine BP values become difficult to interpret when medication exposure, adherence, visit timing, and measurement conditions are incompletely documented. Prospective studies with standardized measurements and verified treatment histories are needed before such monitoring can be evaluated.

## Figures and Tables

**Figure 1:**
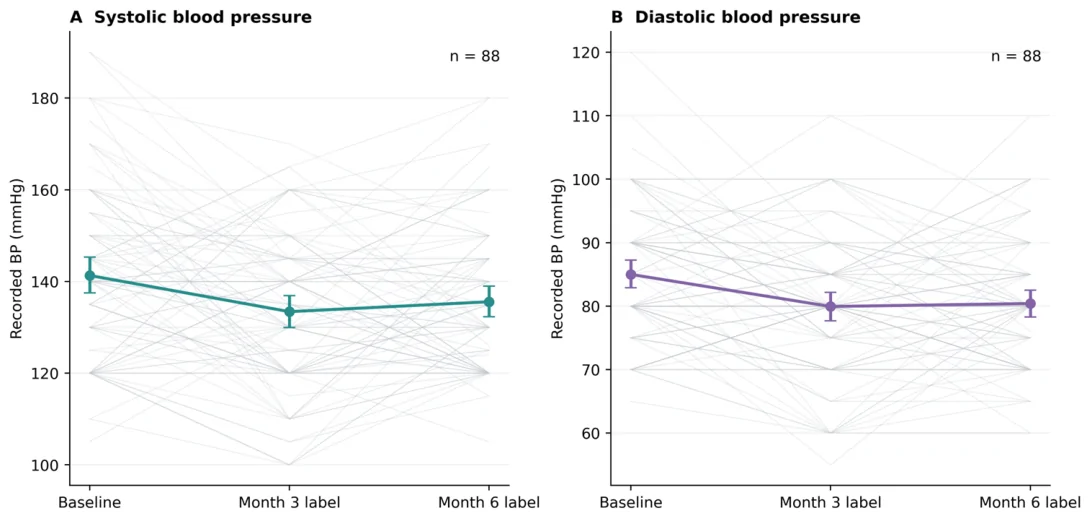
Three-visit recorded blood pressure in the fixed complete subsets (*n* = 88 for each panel). (**A**) Systolic blood pressure (SBP); teal points and lines show arithmetic means. (**B**) Diastolic blood pressure (DBP); purple points and lines show arithmetic means. In both panels, thin gray lines connect individual observations, and bars are participant-bootstrap 95% confidence intervals for the means (10,000 resamples). The connecting lines guide the eye and do not imply a continuous time slope. Month 3 and month 6 are source visit labels.

**Figure 2:**
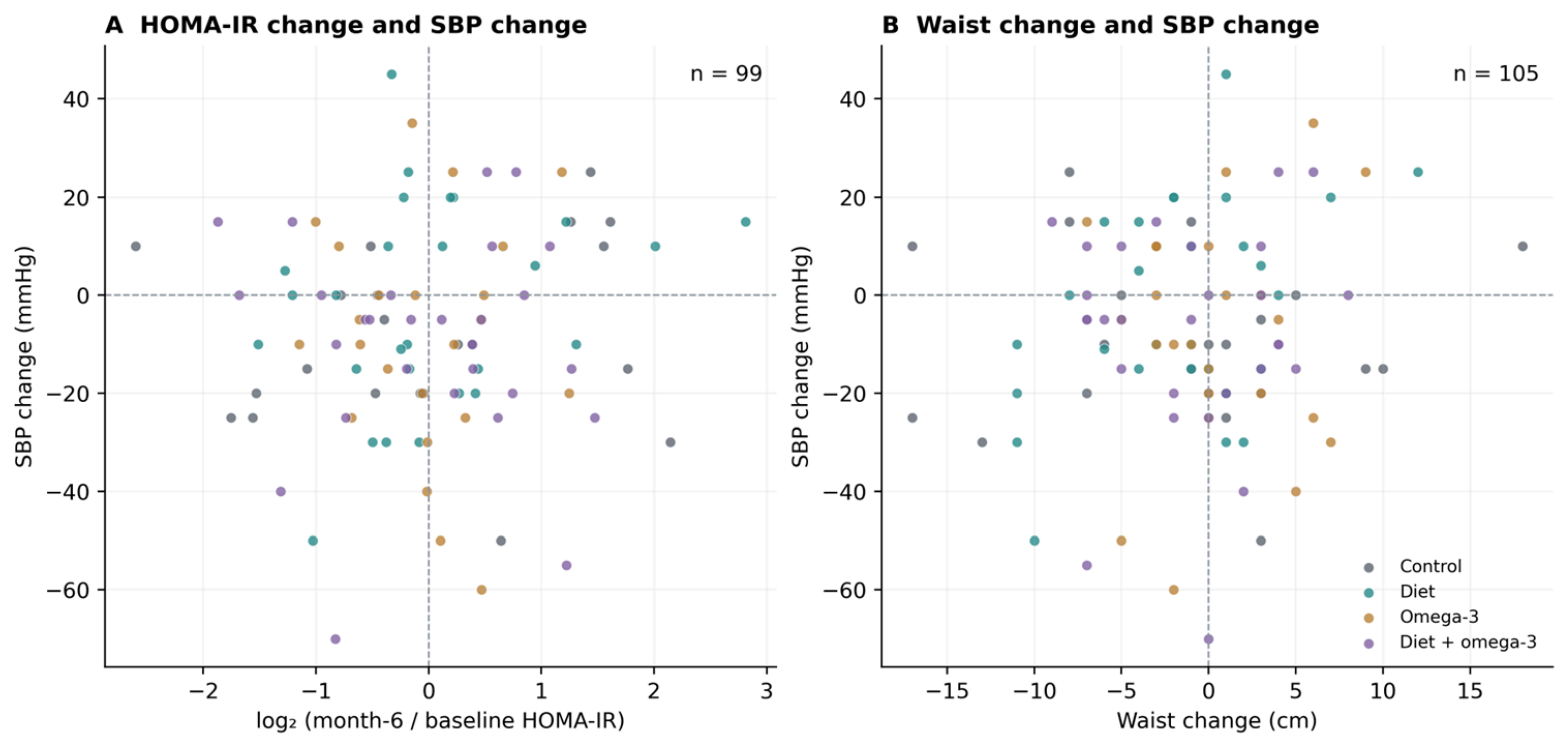
Unadjusted individual changes. Panel (**A**) contains the revised HOMA complete cases without an explicit insulin entry (*n* = 99); panel (**B**) contains the waist model complete cases (*n* = 105). Colors identify nonrandomized baseline prescription categories. Dashed lines indicate no change. A log2 HOMA ratio of +1 represents doubling and −1 halving. Adjusted estimates are reported in Table 5.

**Figure 3:**
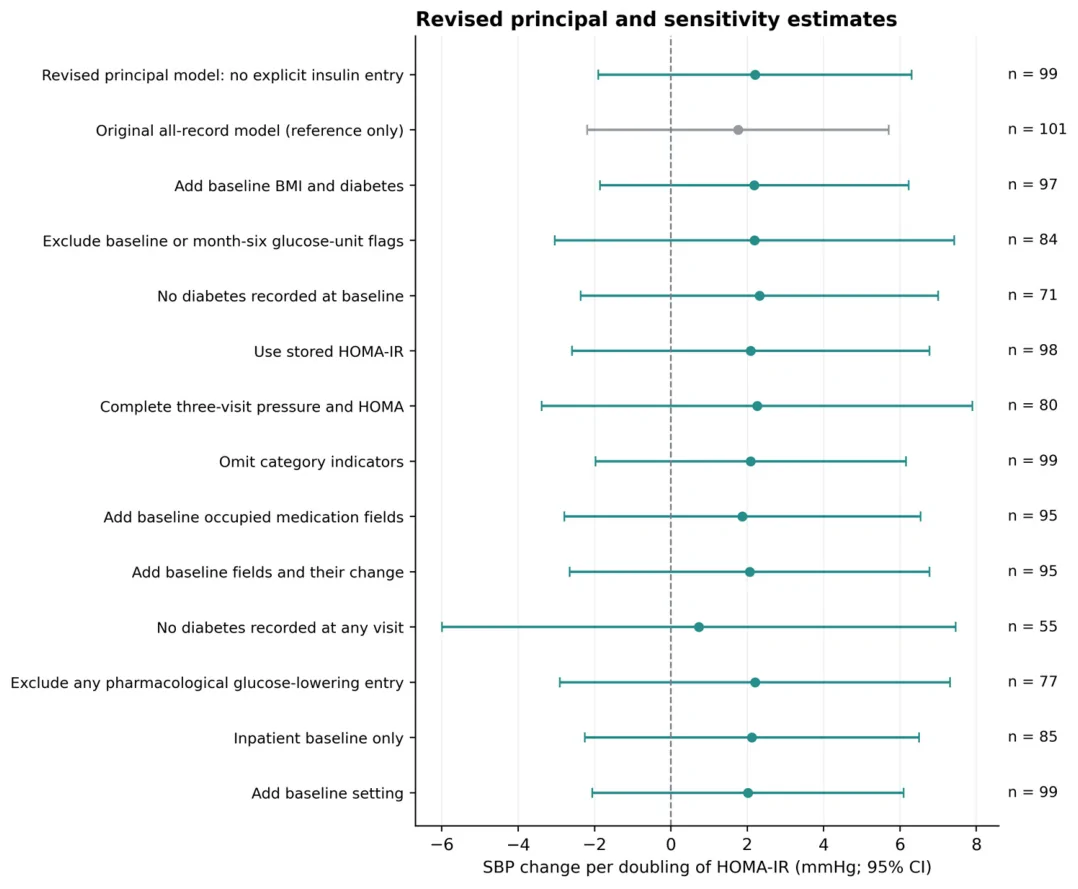
Revised principal and sensitivity HOMA–SBP coefficients per doubling of HOMA-IR, with pointwise HC3 95% CIs. All revised specifications exclude explicit insulin exposure; the gray original all-record estimate is shown only for numerical comparison. The dashed line marks zero. Models include baseline SBP, baseline log2 HOMA, age, sex, and category except where specified. Exact coefficients and denominators are in Table S1.

**Table 1:** Baseline characteristics of the 150-record CFR cohort.

Characteristic	Available *n*	Summary
Age years	150	54.99 ± 10.43
Women	150	71/150 (47.3%)
Recorded hypertension	149	125/149 (83.9%)
Recorded diabetes	149	40/149 (26.8%)
Current smoking	149	29/149 (19.5%)
Baseline BMI kg/m^2^	148	33.98 ± 4.81
Baseline waist cm	149	110.30 ± 11.24
Baseline weight kg	148	96.21 ± 17.43
Baseline SBP mmHg	149	142.21 ± 20.71
Baseline DBP mmHg	149	86.34 ± 11.30
Baseline recalculated HOMA-IR	147	3.32 [2.16–4.88]

Values are mean ± SD, median [IQR], or count/available *n* (%). Categories contained 39 control, 37 diet, 37 omega-3, and 37 combined records. Paired pressure counts were 24, 29, 24 and 31; revised principal-model counts were 21, 27, 23 and 28. Baseline HOMA-IR excludes two participants with any explicit insulin entry.

**Table 2:** Baseline characteristics by prescribed management category.

Characteristic	Control (*n* = 39)	Diet (*n* = 37)	Omega-3 (*n* = 37)	Combined (*n* = 37)	*p*
Age, years	60.10 ± 11.14	55.76 ± 8.27	52.22 ± 10.76	51.59 ± 9.27	0.002
Women	25/39 (64.1%)	17/37 (45.9%)	16/37 (43.2%)	13/37 (35.1%)	0.076
Inpatient at baseline	36/39 (92.3%)	34/37 (91.9%)	31/37 (83.8%)	31/37 (83.8%)	0.482
Recorded hypertension	34/38 (89.5%)	35/37 (94.6%)	32/37 (86.5%)	24/37 (64.9%)	0.003
Recorded diabetes	16/38 (42.1%)	10/37 (27.0%)	7/37 (18.9%)	7/37 (18.9%)	0.076
Current smoking	6/38 (15.8%)	4/37 (10.8%)	10/37 (27.0%)	9/37 (24.3%)	0.261
≥1 antihypertensive field occupied	31/32 (96.9%)	32/37 (86.5%)	26/34 (76.5%)	20/36 (55.6%)	<0.001
Pharmacological glucose-lowering treatment	10/38 (26.3%)	7/37 (18.9%)	4/37 (10.8%)	5/37 (13.5%)	0.301
BMI, kg/m^2^	33.97 ± 4.96	35.45 ± 5.09	33.18 ± 4.41	33.32 ± 4.60	0.129
Waist, cm	110.00 ± 12.64	113.30 ± 11.52	107.65 ± 8.64	110.25 ± 11.43	0.240
Weight, kg	91.46 ± 16.48	101.28 ± 20.06	93.46 ± 11.64	98.84 ± 19.21	0.040
SBP, mmHg	146.71 ± 19.00	140.00 ± 19.90	141.76 ± 20.89	140.27 ± 23.00	0.256
DBP, mmHg	87.76 ± 10.64	86.35 ± 10.38	85.41 ± 11.75	85.81 ± 12.61	0.744
Recalculated HOMA-IR	3.64 [1.85–4.83]	3.15 [2.27–4.46]	3.21 [2.48–4.86]	3.60 [2.12–4.95]	0.806

Values are mean ± SD, median [IQR] or count/available *n* (%). Control denotes usual care; diet, dietary advice; omega-3, supplementation; combined, both recommendations. Assignment was non-random. Antihypertensive fields represent occupied source headings, not validated drug classes; denominators require all five fields. HOMA-IR excludes the two participants with any explicit insulin entry. *p* values are descriptive Kruskal–Wallis or chi-square tests without multiplicity adjustment.

**Table 3:** Baseline-to-month-six changes by prescribed management category.

Outcome	Category	*n*	Mean Change (95% CI)	Adjusted Difference vs. Control (95% CI)	*p*	*q*
SBP, mmHg (global *p* = 0.743)	Control	24	−8.12 (−15.00 to −1.25)	Reference		
	Diet	29	−2.07 (−9.55 to 5.38)	4.00 (−4.87 to 12.87)	0.373	0.980
	Omega-3	24	−8.12 (−17.71 to 0.83)	1.27 (−9.68 to 12.22)	0.819	0.980
	Diet + omega-3	31	−8.23 (−15.81 to −1.45)	0.74 (−8.97 to 10.45)	0.879	0.980
DBP, mmHg (global *p* = 0.584)	Control	24	−6.25 (−11.67 to −0.83)	Reference		
	Diet	29	−3.93 (−7.59 to −0.31)	2.72 (−3.22 to 8.66)	0.366	0.980
	Omega-3	24	−6.88 (−11.88 to −2.50)	0.10 (−6.15 to 6.36)	0.974	0.980
	Diet + omega-3	31	−6.94 (−10.81 to −2.90)	0.11 (−6.20 to 6.42)	0.973	0.980
HOMA-IR, log2 ratio (global *p* = 0.881)	Control	21	−0.01 (−0.56 to 0.54)	Reference		
	Diet	27	0.03 (−0.32 to 0.41)	−0.02 (−0.46 to 0.43)	0.939	0.980
	Omega-3	23	−0.03 (−0.28 to 0.23)	0.16 (−0.37 to 0.69)	0.549	0.980
	Diet + omega-3	28	−0.02 (−0.35 to 0.31)	0.02 (−0.46 to 0.50)	0.944	0.980
Waist, cm (global *p* = 0.700)	Control	24	−1.46 (−4.62 to 1.79)	Reference		
	Diet	28	−1.71 (−3.75 to 0.32)	−0.05 (−4.13 to 4.03)	0.980	0.980
	Omega-3	24	0.46 (−1.12 to 2.12)	1.06 (−3.10 to 5.21)	0.615	0.980
	Diet + omega-3	29	−1.07 (−2.76 to 0.66)	−0.38 (−4.68 to 3.92)	0.862	0.980
Weight, kg (global *p* = 0.883)	Control	22	−1.76 (−4.60 to 0.56)	Reference		
	Diet	28	−1.34 (−2.80 to 0.11)	0.58 (−3.07 to 4.23)	0.755	0.980
	Omega-3	23	−0.14 (−1.86 to 1.59)	1.10 (−2.41 to 4.61)	0.536	0.980
	Diet + omega-3	27	−0.89 (−2.30 to 0.53)	0.31 (−3.33 to 3.95)	0.867	0.980

Changes are month-6-labeled minus baseline values. HOMA change is log2(month-6/baseline) and excludes explicit insulin exposure; 0.1 log2 units correspond to approximately 7% ratio change. Mean-change CIs use 10,000 participant-bootstrap resamples. Adjusted differences come from change regressed on category indicators, the corresponding baseline value, age per decade, and sex, with HC3 residual-df t intervals. The global *p*-value is from an HC3 Wald F test of all three category indicators. Benjamini–Hochberg *q* values cover all 15 contrasts. Categories are nonrandomized baseline prescriptions without measured adherence.

**Table 4:** Interval changes in the fixed three-visit blood pressure subsets.

Pressure	Interval	*n*	Mean Change mmHg	95% CI mmHg	*p*	*q*
SBP	0–3 months	88	−7.90	−12.33 to −3.58	0.001	0.002
SBP	3–6 months	88	2.16	−1.68 to 6.05	0.391	0.469
SBP	0–6 months	88	−5.74	−10.00 to −1.57	0.013	0.019
DBP	0–3 months	88	−5.06	−7.56 to −2.61	<0.001	0.001
DBP	3–6 months	88	0.47	−2.00 to 2.95	0.863	0.863
DBP	0–6 months	88	−4.59	−6.97 to −2.34	<0.001	0.001

Each pressure component uses 88 participants at all three visits. Mean changes are later visit minus earlier visit; CIs are percentile participant-bootstrap intervals (10,000 resamples). Two-sided Wilcoxon *p*-values and Benjamini–Hochberg *q*-values use the six interval contrasts as one family. Fixed-subset visit means are reported in the text and Figure 1.

**Table 5:** Adjusted metabolic change coefficients for SBP and DBP change.

BP	Exposure Change Scale	*n*	Coefficient mmHg	95% CI mmHg	*p*	*q*
SBP	HOMA per doubling	99	2.20	−1.90 to 6.31	0.289	0.434
DBP	HOMA per doubling	99	−0.47	−3.02 to 2.08	0.718	0.718
SBP	Waist per 5 cm	105	3.22	0.57 to 5.87	0.018	0.107
DBP	Waist per 5 cm	105	1.14	−0.88 to 3.15	0.265	0.434
SBP	Weight per kg	100	0.56	−0.23 to 1.35	0.161	0.434
DBP	Weight per kg	100	0.13	−0.35 to 0.61	0.588	0.705

Separate models adjust for baseline pressure, the corresponding baseline metabolic measure, age per decade, sex, and category. HOMA models exclude any explicit insulin entry; anthropometric models use their available complete cases. CIs are pointwise HC3 residual-df t intervals. Benjamini–Hochberg *q* values were recalculated across the six coefficients after revision of the HOMA models. None met *q* < 0.05.

**Table 6:** Descriptive paired pressure-threshold transitions in 108 patients.

Threshold mmHg	Transition	Patients *n*	Percentage
140/90	Below at both visits	31	28.7
140/90	Not below to below	31	28.7
140/90	Below to not below	11	10.2
140/90	Not below at both visits	35	32.4
130/80	Below at both visits	5	4.6
130/80	Not below to below	21	19.4
130/80	Below to not below	9	8.3
130/80	Not below at both visits	73	67.6

Classification as below a threshold requires both SBP and DBP to be strictly lower. For 140/90, 42/108 were below at baseline and 62/108 at month six; exact McNemar *p* = 0.003, *q* = 0.006. For 130/80, corresponding counts were 14/108 and 26/108; *p* = *q* = 0.043. *q* values use the two threshold tests. Categories describe recorded values rather than sustained control, remission, or individualized target attainment.

## Data Availability

The data presented in this study are available on reasonable request from the corresponding authors. The data are not publicly available because they derive from patient clinical records and are subject to privacy and ethical restrictions.

## Supplementary Materials

The following supporting information can be downloaded at: https://www.mdpi.com/article/10.3390/jcm15218496/s1, Table S1: Revised principal, original reference and sensitivity HOMA–SBP estimates; Table S2: Baseline characteristics by paired-pressure availability; Table S3: Contextual baseline-to-month-6 paired changes; Table S4: Revised principal-model inclusion and sequential exclusions; Table S5: Inflammatory-marker availability and zero coding; Table S6: Recorded glucose-lowering, antihypertensive and lipid-lowering treatment by visit; Table S7: Characteristics and BP change by baseline setting; Table S8: Terminal digits of recorded BP values.

## Abbreviations

The following abbreviations are used in this manuscript:

ACE	Angiotensin-converting enzyme
APC	Article processing charge
BMI	Body mass index
BP	Blood pressure
CFR	CF Clinical Hospital Timișoara (Căile Ferate Române, Romanian Railways)
CI	Confidence interval
DASH	Dietary Approaches to Stop Hypertension
DBP	Diastolic blood pressure
DHA	Docosahexaenoic acid
eGFR	Estimated glomerular filtration rate
EPA	Eicosapentaenoic acid
HC3	Heteroscedasticity-consistent covariance estimator type 3
HOMA-IR	Homeostasis model assessment of insulin resistance
IL-6	Interleukin-6
IQR	Interquartile range
SBP	Systolic blood pressure
SD	Standard deviation
SMD	Standardized mean difference
SGLT2	Sodium–glucose cotransporter-2
STROBE	Strengthening the Reporting of Observational Studies in Epidemiology
TNF-α	Tumor necrosis factor-α
