# Supplementary material for: Recalculated HOMA-IR and Recorded Blood Pressure Changes in Treated Internal Medicine Patients: An Exploratory Retrospective Study
Source: J Clin Med. 2026 Nov 1;15(21):8496. doi: 10.3390/jcm15218496 (PMC13764382; doi:10.3390/jcm15218496)
Supplement: Supplementary file 1 [file jcm-15-08496-s001.zip › jcm-4659534-supplementary.pdf]

Supplementary Tables

Recalculated HOMA-IR and Recorded Blood Pressure Changes in Treated Internal Medicine Patients: An Exploratory Retrospective Study

Table S1. Revised principal, original reference and sensitivity HOMA–SBP estimates.

**Specification**	***n***	**Coefficient, mmHg**	**95% CI, mmHg**	***p***
Revised principal model: no explicit insulin entry	99	2.20	−1.90 to 6.31	0.289
Original all-record model (reference only)	101	1.76	−2.19 to 5.71	0.379
Add baseline BMI and diabetes	97	2.18	−1.86 to 6.23	0.286
Exclude baseline or month-six glucose-unit flags	84	2.19	−3.04 to 7.42	0.407
No diabetes recorded at baseline	71	2.32	−2.36 to 7.00	0.325
Use stored HOMA-IR	98	2.09	−2.59 to 6.77	0.377
Complete three-visit pressure and HOMA	80	2.26	−3.38 to 7.90	0.428
Omit category indicators	99	2.09	−1.97 to 6.15	0.309
Add baseline occupied medication fields	95	1.87	−2.79 to 6.54	0.427
Add baseline fields and their change	95	2.06	−2.65 to 6.77	0.387
No diabetes recorded at any visit	55	0.73	−5.99 to 7.46	0.827
Exclude any pharmacological glucose-lowering entry	77	2.20	−2.91 to 7.31	0.393
Inpatient baseline only	85	2.12	−2.26 to 6.50	0.337
Add baseline setting	99	2.02	−2.06 to 6.09	0.328

Coefficients are mmHg per doubling of HOMA-IR, with pointwise HC3 residual-df t intervals and nominal two-sided p values. All revised specifications exclude any explicit insulin entry at any visit. The original all-record model includes two such records and is retained only for numerical comparison, without a validated insulin-resistance interpretation. The insulin-exclusion revision and all restrictions were made after data access. No recorded baseline diabetes and no pharmacological glucose-lowering entry are key interpretability restrictions, not proof of insulin-naive status or absence of insulin antibodies. Models include baseline SBP, baseline log2 HOMA, age, sex and category unless specified. Stored-HOMA models use stored rather than recalculated baseline and change terms. Medication counts represent occupied source fields, not verified regimens or doses; conditioning on their change can introduce bias. Similar estimates cannot validate HOMA or establish absence of confounding.

Table S2. Selected baseline characteristics by paired-pressure availability.

**Characteristic**	**Paired pressure records n and value**	**Other source records n and value**	***p***
Age, years	108; 55.75 ± 10.03	42; 53.02 ± 11.27	0.063
Baseline SBP, mmHg	108; 141.94 ± 20.45	41; 142.93 ± 21.62	0.722
Baseline DBP, mmHg	108; 85.88 ± 11.31	41; 87.56 ± 11.30	0.335
Baseline BMI, kg/m²	106; 33.88 ± 4.84	42; 34.25 ± 4.80	0.677
Baseline recalculated HOMA-IR	106; 3.49 [2.15–4.95]	41; 3.16 [2.23–4.14]	0.331

Paired-pressure group contains 108 source records; the remaining group contains 42. Values use available baseline entries, with mean ± SD except HOMA median [IQR]. p values are two-sided Mann–Whitney tests, descriptive and unadjusted. These comparisons cannot establish random

missingness. This comparison concerns paired-pressure availability (108 versus 42), not revised principal-model inclusion (99 versus 51). It cannot exclude selection related to unobserved outcomes or establish random missingness. The direct 99-versus-51 comparison is now provided in Table S4. HOMA summaries exclude the two participants with any explicit insulin entry.

Table S3. Contextual baseline-to-month-6-labeled paired changes.

Variable	*n*	Mean change	95% CI	*p*	*q*
SBP, mmHg	108	−6.53	−10.55 to −2.73	0.003	0.008
DBP, mmHg	108	−5.96	−8.14 to −3.81	<0.001	<0.001
HOMA-IR, units	99	−0.19	−0.86 to 0.53	0.481	0.481
Waist, cm	105	−0.98	−2.07 to 0.10	0.085	0.107
Weight, kg	100	−1.04	−1.95 to −0.15	0.062	0.103

Mean changes are month-6-labeled minus baseline values. CIs are percentile participant-bootstrap intervals (10,000 resamples; seed 82110). p values are two-sided Wilcoxon signed-rank tests, which concern a different estimand from the mean-change CIs. q values cover the five paired comparisons. HOMA-IR excludes any explicit insulin entry; other variables use their available pairs.

Table S4. Revised principal-model inclusion and sequential exclusions.
The revised principal model includes 99/150 records; 51 were not analyzed. Sequential mutually exclusive reasons were missing paired baseline/month-6 SBP (42), missing paired glucose–insulin inputs among the remaining records (7), and explicit insulin exposure among otherwise complete records (2). The final exclusion concerns HOMA interpretability, not missingness. No further records were lost to covariates. Differences are descriptive and cannot establish random missingness or unbiased selection. SMD is the mean difference divided by the square root of the mean of the two variances. Baseline HOMA is shown as a numerical mean ± SD for this audit; the excluded group includes two insulin-exposed records and its HOMA values are not interpreted as valid insulin-resistance estimates.

Variable	Included (n = 99)	Other records (n = 51)	SMD
Age years	99; 55.53 ± 10.33	51; 53.94 ± 10.63	0.151
SBP mmHg	99; 142.98 ± 20.61	50; 140.70 ± 21.02	0.110
DBP mmHg	99; 86.57 ± 11.51	50; 85.90 ± 10.96	0.059
BMI kg/m2	97; 34.11 ± 4.83	51; 33.74 ± 4.81	0.076
Recalculated HOMA-IR	99; 4.28 ± 3.10	50; 3.80 ± 2.50	0.169
Women	46/99 (46.5%)	25/51 (49.0%)	—
Recorded diabetes	28/99 (28.3%)	12/50 (24.0%)	—

Table S5. Inflammatory-marker availability.

Counts describe source entries, not clinically verified normal or abnormal inflammatory states. Units, detection limits and numeric-zero meaning were unavailable in the workbook. Positive means a numeric entry >0, not necessarily above a clinical reference range. Missing entries were not coded zero. Revised principal-model baseline availability was 84/99 for TNF-α and 83/99 for IL-6. These fields were not used for validated inflammatory adjustment.

Marker and visit	Present	Positive	Numeric zero	Missing
TNF-α baseline	125	107	18	25
IL-6 baseline	121	58	63	29
TNF-α month 3	85	84	1	65
IL-6 month 3	87	51	36	63
TNF-α month 6	93	91	2	57
IL-6 month 6	91	51	40	59

Table S6. Recorded glucose-lowering, antihypertensive and lipid-lowering treatment by visit.

Panel A: diabetes-treatment field	Baseline	Month 3	Month 6
Field documented	149	105	108
No glucose-lowering entry	108	75	73
Diet only	15	10	11
Any pharmacological entry	26	20	24
Metformin[a]	20	14	16
Sulfonylurea[a]	10	8	9
Thiazolidinedione	1	0	0
Insulin	1	1	2
Other or unspecified entry	1	1	2
Incretin-based therapy or SGLT2 inhibitor	0	0	0

Counts of patients among those with the free-text diabetes-treatment field documented at each visit. Entries were coded as written: metformin (Siofor, alone or combined), sulfonylurea (glimepiride, glibenclamide), thiazolidinedione (rosiglitazone), insulin, diet only, and other or unspecified entries (a non-classifiable product name; a numeric code "1"). [a] The glibenclamide–metformin combination (n = 4 at baseline) is counted in both classes, so classes are not mutually exclusive. Two patients had an insulin entry at any visit. Among 108 patients with the field documented at baseline and month six, recorded drug classes changed in four (diet only → metformin; none → metformin; metformin → insulin; none → unspecified entry). Two month-six entries (glibenclamide–metformin; metformin) had been recorded in the adjacent glucose-tolerance field and were reassigned to the treatment field. Dose and the timing of the last dose relative to fasting sampling were not recorded.

Panel B: medication heading occupied	Baseline	Month 3	Month 6
Beta-blocker	63/142 (44.4%)	54/105 (51.4%)	53/107 (49.5%)
ACE inhibitor	79/143 (55.2%)	61/105 (58.1%)	62/108 (57.4%)
Calcium-channel blocker	24/142 (16.9%)	19/105 (18.1%)	20/106 (18.9%)
Diuretic	45/143 (31.5%)	35/104 (33.7%)	36/108 (33.3%)
Angiotensin receptor blocker	13/140 (9.3%)	8/105 (7.6%)	12/108 (11.1%)
Statin	28/143 (19.6%)	29/105 (27.6%)	28/108 (25.9%)
Fibrate	7/143 (4.9%)	6/105 (5.7%)	8/107 (7.5%)
Occupied antihypertensive fields, median [IQR]	2 [1–2]	2 [1–2]	2 [1–2]

Occupied means a nonblank entry other than zero in the source heading; denominators are records with that heading documented. Headings were free text with inconsistent spelling and some apparent class-placement errors, so occupancy does not equal a validated drug class, and doses were not recorded. Among 102 patients with all five antihypertensive headings documented at baseline and month six, the occupied-field count decreased in 6, was unchanged in 82 and increased in 14; an unchanged count does not imply unchanged drugs or doses.

Table S7. Baseline characteristics and blood pressure change by baseline setting.

Characteristic	Inpatient (*n* = 132)	Outpatient (*n* = 18)	*p*
Age, years	55.90 ± 10.00	48.28 ± 11.29	0.012
Women	60/132 (45.5%)	11/18 (61.1%)	0.314
Recorded hypertension	114/132 (86.4%)	11/17 (64.7%)	0.034
Recorded diabetes	38/132 (28.8%)	2/17 (11.8%)	0.159
Recorded impaired glucose tolerance	20/132 (15.2%)	1/17 (5.9%)	0.469
Current smoking	27/132 (20.5%)	2/17 (11.8%)	0.527
≥1 antihypertensive field occupied	101/125 (80.8%)	8/14 (57.1%)	0.079
Pharmacological glucose-lowering treatment	25/132 (18.9%)	1/17 (5.9%)	0.308
BMI, kg/m²	33.95 ± 4.82	34.24 ± 4.90	0.810
Waist, cm	110.47 ± 11.53	109.06 ± 9.08	0.722
Baseline SBP, mmHg	140.98 ± 20.53	151.76 ± 20.15	0.031
Baseline DBP, mmHg	85.80 ± 11.19	90.59 ± 11.58	0.088
Recalculated HOMA-IR, median [IQR]	3.22 [2.13–4.85]	3.77 [2.36–4.95]	0.563
SBP change to month six, mmHg (n)	−4.41 ± 19.60 (94)	−20.71 ± 23.36 (14)	0.028
DBP change to month six, mmHg (n)	−4.99 ± 11.40 (94)	−12.50 ± 11.22 (14)	0.031
Revised principal-model records	85	14	

Inpatient status was identified by an inpatient observation-chart number at the baseline visit; all follow-up visits were outpatient visits. Values are mean ± SD, median [IQR] or count/available n (%). p values are descriptive two-sided Mann–Whitney or Fisher exact tests. Reasons for admission were not available as a structured field and could not be retrieved; recorded diagnoses are shown. The larger decrease among outpatients coincided with higher baseline values; neither setting effects nor regression to the mean can be identified separately. HOMA summaries exclude the two participants with any explicit insulin entry.

Table S8. Terminal digits of recorded blood pressure values.

Component and visit	*n*	Ending in 0	Ending in 5	Other final digit	Multiple of 5
SBP baseline	149	117 (78.5%)	32 (21.5%)	0 (0.0%)	100.0%
DBP baseline	149	106 (71.1%)	43 (28.9%)	0 (0.0%)	100.0%
SBP month 3	105	74 (70.5%)	31 (29.5%)	0 (0.0%)	100.0%
DBP month 3	105	68 (64.8%)	36 (34.3%)	1 (1.0%)	99.0%
SBP month 6	108	75 (69.4%)	31 (28.7%)	2 (1.9%)	98.1%
DBP month 6	108	72 (66.7%)	35 (32.4%)	1 (0.9%)	99.1%
All values	724	512 (70.7%)	208 (28.7%)	4 (0.6%)	99.4%

Counts concern final digits of chart entries, not archived device output. The distribution departs strongly from uniform terminal digits (chi-square goodness-of-fit across ten digits, $p < 0.001$). This supports pronounced digit preference but cannot identify whether it arose from rounding, manual measurement, averaging or transcription. Values were analyzed as recorded.
